# CMOS-compatible dense arrays of Ge quantum dots on the Si(001) surface: hut cluster nucleation, atomic structure and array life cycle during UHV MBE growth

**DOI:** 10.1186/1556-276X-6-345

**Published:** 2011-04-15

**Authors:** Larisa V Arapkina, Vladimir A Yuryev

**Affiliations:** 1 A. M. Prokhorov General Physics Institute of RAS, 38 Vavilov Street, Moscow, 119991, Russia

## Abstract

We report a direct observation of Ge hut nucleation on Si(001) during UHV molecular beam epitaxy at 360°C. Nuclei of pyramids and wedges were observed on the wetting layer (WL) (*M *× *N*) patches starting from the coverage of 5.1 Å and found to have different structures. Atomic models of nuclei of both hut species have been built as well as models of the growing clusters. The growth of huts of each species has been demonstrated to follow generic scenarios. The formation of the second atomic layer of a wedge results in rearrangement of its first layer. Its ridge structure does not repeat the nucleus. A pyramid grows without phase transitions. A structure of its vertex copies the nucleus. Transitions between hut species turned out to be impossible. The wedges contain point defects in the upper corners of the triangular faces and have preferential growth directions along the ridges. The derived structure of the {105} facet follows the paired dimer model. Further growth of hut arrays results in domination of wedges, and the density of pyramids exponentially drops. The second generation of huts arises at coverages *>*10 Å; new huts occupy the whole WL at coverages ~14 Å. Nanocrystalline Ge 2D layer begins forming at coverages *>*14 Å.

## Introduction

Development of CMOS-compatible processes of formation of germanium quantum dot (QD) dense arrays on the (001) silicon surface as well as multilayer Ge/Si epitaxial heterostructures on their basis is a challenging task of great practical significance [1-14]. An important direction of applied researches in this area is the development of highly efficient monolithic far and mid infrared detector arrays which could be produced by a standard CMOS technology [9-14]. Such detectors have to combine high perfection (uniformity, sensitivity, operating life, etc.) with high yield and low production price. A requirement of CMOS compatibility of technological processes imposes a hard constraint on conditions of all the phases of the QD array manufacturing starting from the stage of preparation of a clean Si surface for Ge/Si heterostructure deposition: on the one hand, formation of a photosensitive layer must be one of the latest operations of the whole device production cycle because otherwise the structure with QDs would be destroyed by further high-temperature annealings; on the other hand, high temperature processes during Ge/Si heterostructure formation on the late phase of the detector chip production would certainly wreck the readout circuit formed on the crystal. Therefore, lowering of the array formation temperature down to the values of ≲ 450°C^a ^is strongly required [1,11], and the Ge QD arrays meeting this requirement are referred to as CMOS-compatible ones.

In addition to the requirement of the low temperature of a Ge QD array formation, both high density of the germanium nanoclusters (*>*10^11 ^cm^-2^) and high uniformity of the cluster shapes and sizes (dispersion *<*10%) in the arrays are necessary for employment of such structures in CMOS IR detectors [12]. The molecular beam epitaxy (MBE) is known to be the main technique of formation of Ge/Si heterostructures with QDs [2,15]. A high density of the self-assembled hut clusters can be obtained in the MBE process of the Ge/Si(001) structure formation when depositing germanium on the Si(001) substrate heated to a temperature *T*_gr _≲ 550°C. In this case, the lower the temperature of the silicon substrate during the Ge deposition the higher the density of the clusters at the permanent quantity of the deposited Ge [16,17]. For example, the density of the Ge clusters in the array was 6 × 10^11 ^cm^-2 ^at *T*_gr _= 360°C, and the effective thickness of the deposited germanium layer^b ^*h*_Ge _= 8 Å; the cluster density of only ~2 × 10^11 ^cm^-2 ^was obtained at *T*_gr _= 530°C and the same value of *h*_Ge _[18].

There is another approach for obtaining dense cluster arrays. The authors of Refs. [4,19-21] reached the cluster density of ~9 × 10^11 ^cm^-2 ^using the pulsed irradiation of the substrate by a low-energy Ge^+ ^ion beam during the MBE growth of the Ge/Si(001) heterostructures at *T*_gr _as high as 570°C.

Obtaining of the arrays of the densely packed Ge QDs on the Si(001) surface is an important task, but the problem of formation of uniform arrays of the Ge clusters is much more challenging one. The process of Ge/Si(001) heterostructure formation with the Ge QD dense arrays and predetermined electrophysical and photoelectric parameters cannot be developed until both of these tasks are solved. The uniformity of the cluster sizes and shapes in the arrays determines not only the widths of the energy spectra of the charge-carrier bound states in the QD arrays [4], but in a number of cases the optical and electrical properties of both the arrays themselves and the device structures produced on their basis [22]. To find an approach to the improvement of the Ge QD array uniformity on the Si(001) surface, it is necessary to carry out a detailed morphological investigation of them.

This article presents the results of our recent investigations of several important issues of the Ge dense array formation and growth. We have studied the array nucleation phase (the transition from 2D growth of the wetting layer (WL) to 3D formation of the QD array when the nuclei of both species of huts--pyramids and wedges [18]--begin to arise on the (*M *× *N*) patches of WL)[23]. We have identified by STM the nuclei of both species, determined their atomic structures [18,24] and observed the moment of appearance the first generation of the nuclei. We have investigated with high spatial resolution the peculiarities of each species of huts and their growth and derived their atomic structures [24,25]. We have concluded that the wedge-like huts form because of a phase transition reconstructing the first atomic step of the growing cluster when dimer pairs of its second atomic layer stack up; the pyramids grow without such phase transitions. In addition, we have come to conclusion that wedges contain vacancy-type defects on the penultimate terraces of their triangular facets [24] which may decrease the energy of addition of new atoms to these facets and stimulate the quicker growth on them than on the trapezoidal ones and rapid elongation of wedges. We have shown also comparing the structures and growth of pyramids and wedges that shape transitions between them are very unlikely [24,25]. Finally, we have explored the array evolution during MBE right up to the end of its life when most of clusters coalesce and start forming a nanocrystalline 2D layer.

In the next sections, we present these results in detail.

## Methods, equipment and conditions of experiments

The experiments were made using an integrated ultrahigh vacuum instrument [18] built on the basis of the Riber surface science center with the EVA 32 MBE chamber connected to the STM GPI-300 ultrahigh vacuum scanning tunnelling microscope [26-28]. This equipment allows us to carry out the STM study of samples at any phase of a substrate surface preparation and MBE growth. The samples can be transferred into the STM chamber for the examination and moved back into the MBE vessel for further processing as many times as required never leaving the UHV ambient and preserving the required cleanness for STM investigations with atomic resolution and MBE growth.

Initial substrates were 8 × 8 mm^2 ^squares cut from the specially treated commercial B-doped CZ Si(100) wafers (*p*-type, ρ = 12 Ωcm). After washing and chemical treatment following the standard procedure described elsewhere [29,30] (which included washing in ethanol, etching in the mixture of HNO_3 _and HF and rinsing in the deionized water), the silicon substrates were mounted on the molybdenum STM holders and inflexibly clamped with the tantalum fasteners. The STM holders were placed in the holders for MBE made of molybdenum with tantalum inserts. Then, the substrates were loaded into the airlock and transferred into the preliminary annealing chamber where they were outgassed at the temperature of around 565°C and the pressure of about 5 × 10^-9 ^Torr for about 24 h. Eventually, the substrates were moved for final treatment into the MBE chamber evacuated down to about 10^-11 ^Torr. There were two stages of annealing in the process of substrate heating in the MBE chamber--at 600°C for 5 min and at 800°C for 3 min [18]. The final annealing at the temperature greater than 900°C was carried out for nearly 2.5 min with the maximum temperature of about 925°C (1.5 min). Then, the temperature was rapidly lowered to about 750°C. The rate of the further cooling was around 0.4°C/s that corresponded to the 'quenching' mode applied in [30]. The pressure in the MBE chamber grew to nearly 2 × 10^-9 ^Torr during the deoxidization process. The surfaces of the silicon substrates were completely purified of the oxide film as a result of this treatment; more data on the morphology of the prepared Si(001) clean surfaces can be found in Refs. [29-31].

Ge was deposited directly on the deoxidized Si(001) surface from the source with the electron beam evaporation.^c ^The Ge deposition rate was about 0.15 Å/s; the effective Ge film thickness *h*_Ge _was varied from 4 to 15 Å for different samples. The deposition rate and *h*_Ge _were measured using the XTC film thickness monitor equipped with the graduated in-advance quartz sensor installed in the MBE chamber. The substrate temperature *T*_gr _was 360°C during Ge deposition; the pressure in the MBE chamber did not exceed 10^-9 ^Torr. The rate of the sample cooling down to the room temperature was approximately 0.4°C/s after the deposition.

The samples were heated by Ta radiators from the rear side in both preliminary annealing and MBE chambers. The temperature was monitored with chromel-alumel and tungsten-rhenium thermocouples in the preliminary annealing and MBE chambers, respectively. The thermocouples were mounted in vacuum near the rear side of the samples and *in situ *graduated beforehand against the IM-PAC IS 12-Si pyrometer which measured the sample temperature through chamber windows. The atmosphere's composition in the MBE camber was monitored using the SRS RGA-200 residual gas analyser before and during the process.

After Ge deposition and cooling, the prepared samples were moved for analysis into the STM chamber in which the pressure did not exceed 10^-10 ^Torr. The STM tip was *ex situ *made of the tungsten wire and cleaned by ion bombardment [32] in a special UHV chamber connected to the STM one. The images were obtained in the constant tunnelling current (*I*_t_) mode at the room temperature. The STM tip was zero-biased while the sample was positively or negatively biased (*U*_s_) when scanned in empty- or filled-states imaging mode.

Original firmware [26-28] was used for data acquisition; the STM images were processed afterwards using the WSxM software [33].

## Experimental data and structural models

### Array and hut cluster nucleation

**Figure 1:**
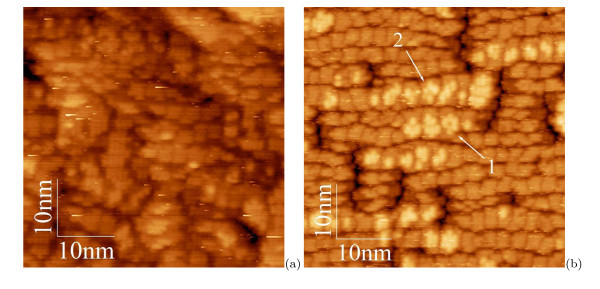
**STM images of Ge WL on Si(001)**: **(a) **before cluster nucleation, *h*_Ge _= 4.4 Å (*U*_s _= -1.86 V, *I*_t _= 100 pA); **(b) **arising nuclei of pyramidal (1) and wedgelike (2) huts, *h*_Ge _= 5.1 Å (*U*_s _= +1.73 V, *I*_t _= 150 pA).

While investigating an evolution of the hut arrays, we have arrived at a conclusion that a moment of an array nucleation during MBE precedes a moment of formation of the first hut on the WL.^d ^It is not a paradox. Hut cluster arrays nucleate when the first hut nuclei arise on the (*M *× *N*) patch of the WL. This process is illustrated in Figure 1. An image (a) demonstrates a typical STM micrograph of the WL with the (*M *× *N*)-patched structure (*h*_Ge _= 4.4 Å). This image does not demonstrate any feature which might be interpreted as a hut nucleus [24]. Such features first arise at the coverages of ~5 Å: they are clearly seen in the image (b), which demonstrates a moment of the array birth (*h*_Ge _= 5.1 Å), and numbered by '1' for the pyramid nucleus, and '2' for the wedge one (several analogous formations can be easily found by the readers on different patches). However, no hut clusters are seen in this picture.

Our interpretation is based on the results reported in Ref. [24] which evidenced that there are two different types of nuclei on Ge WL, which evolve in the process of Ge deposition to pyramidal and wedge-like hut clusters. Having assumed that nuclei emerge on WL as combinations of dimer pairs and/or longer chains of dimers in epitaxial configuration [34] and correspond to the known structure of apexes specific for each hut species [18,25], we have investigated WL patches, one monolayer (ML) high formations on them and clusters of different heights (number of steps) over WL. As a result, we succeeded to select two types of formations different in symmetry and satisfying the above requirements, which first appear at a coverage of ~5 Å and then arise on WL during the array growth. We have interpreted them as hut nuclei, despite their sizes being much less than those predicted by the first principle calculations [35], and traced their evolution to huts.

**Figure 2:**
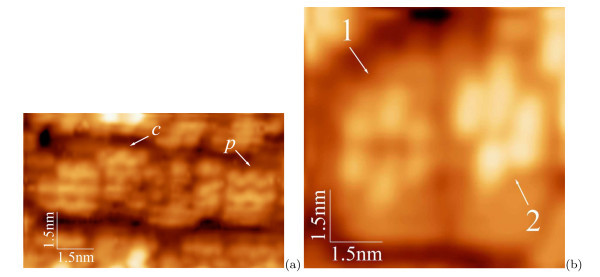
**STM images of Ge WL on Si(001)**: **(a) ***c*(4 × 2) (*c*) and *p*(2 × 2) (*p*) reconstructions within the (*M *× *N*) patches, *h*_Ge _= 6*.*0 Å, *U*_s_ = +1.80 V, *I*_t _= 80 pA; **(b) **new formations arise on the (*M *× *N*) patches due to nucleation of Ge pyramid (1) and wedge (2), *h*_Ge _= 6,0 Å, *U*_s _= +2.60 V, *I*_t _= 80 pA.

**Figure 3:**
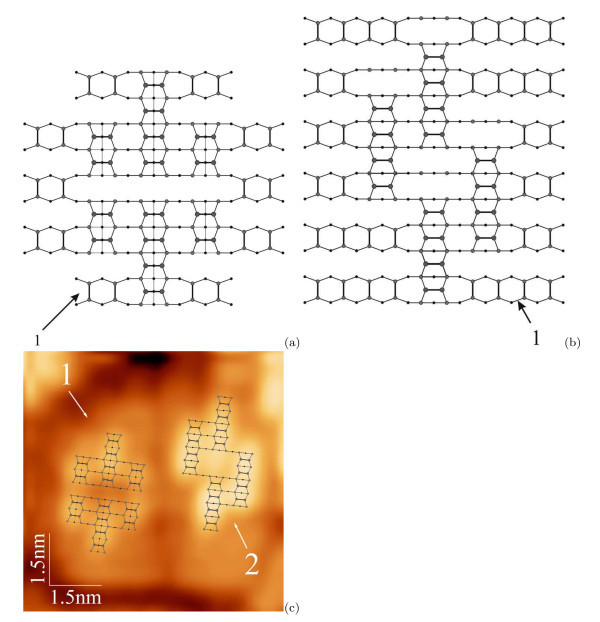
**Models of nuclei of Ge hut clusters corresponding to the images given in Fig. 2b**: **(a) **a pyramid, **(b) **a wedge [1 is the WL in the plots **(a) **and **(b)**]; **(c) **the models superimposed on the image given in Fig. 2b, the numbering is the same as in Fig. 2b.

The nuclei formation is illustrated by Figure 2. The surface structure of the (*M × N*) patches is shown in the micrograph (a). The letter '*c*' indicates the *c*(4 *× *2) reconstructed patch, '*p*' shows a patch with the *p*(2 × 2) reconstruction [36,37]. Both reconstructions are always detected simultaneously, which implies they are very close (or degenerate) by energy. The image (b) shows two adjacent patches reconstructed by the born nuclei: '1' and '2' denote the pyramid (a formation resembling a blossom) and wedge nuclei, respectively [24]. Their structural models derived from many STM images [18,24,25] are presented in Figure 3a, b and superimposed on the images of the nuclei in Figure 3c. Note that both types of nuclei arise at the same moment of the MBE growth. It means that they are degenerate by the formation energy. An issue why two different structures, rather than one, arise to relieve the WL strain [18] remains open, however.

It is necessary to remark here that the nuclei are always observed to arise on sufficiently large WL patches. There must be enough room for a nucleus on a single patch. A nucleus cannot be housed on more than one patch. Hence, cluster nucleation is impossible to occur on little (too narrow or short) patches (Figure 2b).

**Figure 4:**
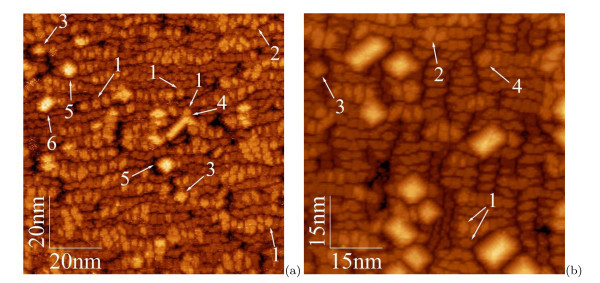
**STM images of Ge WL on Si(001)**: **(a) ***h*_Ge _= 5.4 Å (*U*_s _= +1.80 V, *I*_t _= 100 pA) and **(b) ***h*_Ge _= 6.0 Å (*U*_s _= +2.50 V, *I*_t _= 80 pA). Examples of characteristic features are numbered as follows: nuclei of pyramids (1) and wedges (2) [1 ML high over WL], small pyramids (3) and wedges (2) [2 ML high over WL, a Γ-like wedge [18] is observed in the image (a)], 3 ML high pyramids (5) and wedges (6).

The hut nucleation goes on during the array further evolution. Figure 4 illustrates this process. An array shown in Figure 4a (*h*_Ge _= 5.4 Å) consists of 1-ML nuclei ('1' and '2'), 2-ML and 3-ML pyramids and wedges ('3' and '5', '4' and '6' mark pyramids and wedges, respectively).^e ^Figure 4b (*h*_Ge _= 6.0 Å) demonstrates the simultaneous presence of nuclei ('1' and '2') and 2 ML huts ('3' and '4') with the growth of much higher clusters.

Hut cluster nucleation on the WL surface continues until the final phase of the array life. This peculiarity distinguishes low-temperature growth mode from the high-temperature one [18].

### Structural models

**Figure 5:**
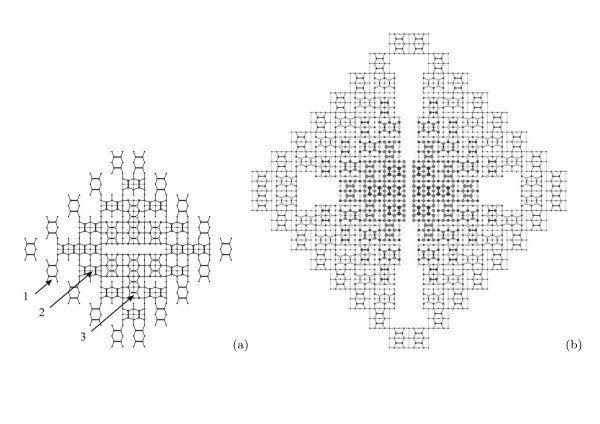
**Top views of the pyramidal QDs consisting of 2 and 6 monoatomic steps and (001) terraces on the WL**: **(a) **2 and **(b) **6 terraces; 1, 2 and 3 designate WL, the first and the second layers of the clusters, respectively.

It is commonly adopted that the hut clusters grow by successively filling the (001) terraces of the {105} faces by the dimer rows [38]. However, formation of the sets of steps and terraces requires the hut base sides to be parallel to the *<*100*>*directions. The pyramid nucleus satisfies this requirement, its sides align with *<*100*>*. Thus, the pyramids grow without phase transition when the second and subsequent layers are added (Figure 5). Only nucleus-like structures of their apexes are rotated 90° with respect to the rows on previous terraces to form the correct epitaxial configuration when the heights are increased by 1 ML, but this rotation does not violate the symmetry of the previous layers of the cluster.

**Figure 6:**
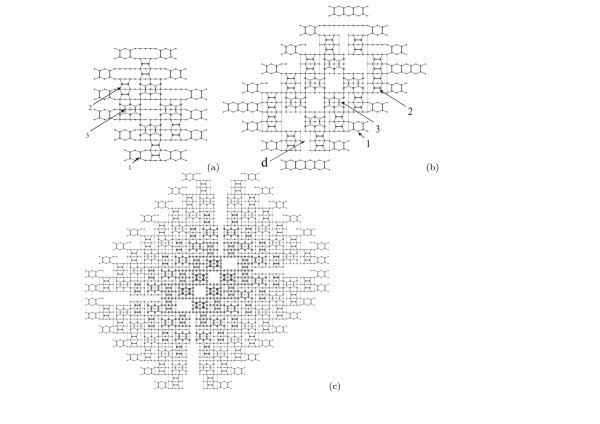
**Growth of a wedge-like cluster**: **(a) **reconstruction of the first layer of a forming wedge during addition of epi-oriented dimer pairs of the second (001) terrace; plots of atomic structures of a Ge wedge-shaped hut cluster composed by **(b) **2 and **(c) **6 monoatomic steps and (001) terraces on the WL (the numbering is the same as in Fig. 5; *d *marks a defect that has arisen because of one translation uncertainty of the left dimer pair position).

A different scenario of growth of the wedge-like clusters has been observed. Two base sides of the wedge nucleus do not align with *<*100*>*(Figure 3b). The ridge structure of a wedge is different from the nucleus structure presented in Figure 3b[18,24,25]. It was shown in Ref. [24] that the structure of the wedge-like cluster arises because of rearrangement of rows of the first layer in the process of the second-layer formation (Figure 6a). The phase transition in the first layer generates the base with all sides directed along the *<*100*>*axes which is necessary to give rise to the {105}-faceted cluster. After the transition, the elongation of the elementary structure is possible only along a single axis which is determined by the symmetry (along the arrows in Figure 6a). A formed 2-ML wedge is plotted in Figure 6b. A structure of the 6-ML wedge appearing as a result of further in-height growth is shown in Figure 6c. The ridge structures of the 2-ML and 6-ML wedges are seen to coincide, which is not the case for different cluster heights. A complete set of the wedge ridges for different cluster heights can be obtained by filling the terraces by epi-oriented pairs of dimers.

It should be noted also that, according to the proposed model, the wedge-like clusters always contain point defects on the triangular (short) facets. The defects are located in the upper corners of the facets and caused by uncertainty of one translation in the position of a dimer pair which forms the penultimate terrace of the triangular facet (Figure 6). The predicted presence of these defects removes the degeneracy of the facets, and hence, an issue of the pyramid symmetry violation which occurs if the pyramid-to-wedge transition is assumed (this issue was discussed in detail in Ref. [18]). In addition, the vacancy-type defects may decrease the energy of addition of new atoms to the triangular facets and stimulate the quicker growth on them than on the trapezoidal ones and rapid elongation of wedges. These defects are absent on the facets of the pyramidal huts. Their triangular facets are degenerate. Therefore, as it follows from our model, the trapezoidal and triangular facets of the wedge are not degenerate with respect to one another even at the very beginning of cluster growth. The wedges can easily elongate by growing on the triangular facets faster than on trapezoidal ones. Pyramids, having degenerate facets, cannot elongate and can grow only in height outrunning wedges. This explains greater heights of pyramids [18].

Analysing the deduced structural models of pyramids and wedges, as well as their behaviour during the array nucleation and growth, we have concluded that shape transitions between the clusters of different species are prohibited [18,24,25].

### Facets

**Figure 7:**
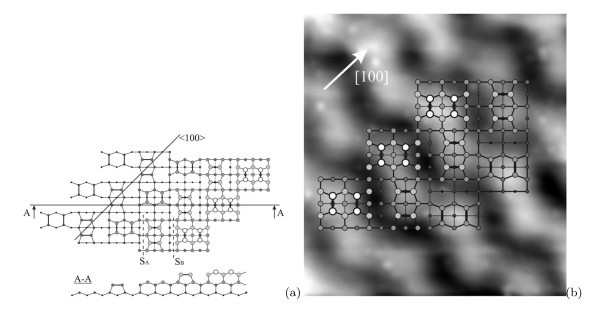
**A structure of hut facets**: **(a) **a structural model of the {105} facet of hut clusters derived from the plots given in Figs. 5 and 6 corresponds to the PD (pairs of dimers) model [39], S_A _and S_B _are commonly adopted designations of the monoatomic steps [45]: atoms situated on higher terraces are shown by larger circles. **(b) **The schematic of the facet superimposed on its STM image (4.3 *× *4.4 nm, *U*_s_ = +3.0 V, *I*_t _= 100 pA): the [100] direction is parallel to the corresponding base side, the steps rise from the lower right to the upper left corner.

The presented models allowed us to deduce a structure of the {105} facets (Figure 7a). This model resulting from the above simple crystallographic consideration corresponds to the paired dimers (PD) [39] rather than more recent rebonded step (RS) model [40,41] which is now believed to be an improvement over the previous PD model proposed by Mo et al.

**Figure 8:**
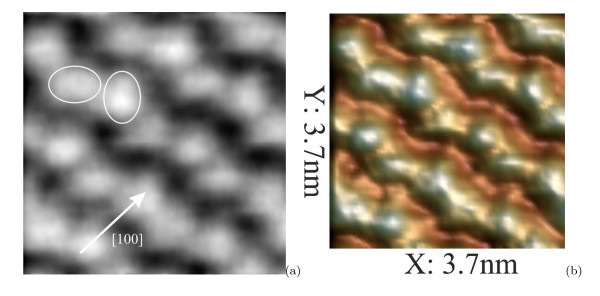
**STM 2D and 3D images of the same area on a Ge hut cluster facet**: **(a) **2D micrograph and **(b) **3D topograph; *h*_Ge _= 10Å, *T*_gr _= 360°C, *U*_s _= +2.1 V, *I*_t _= 80 pA. The sides of the cluster base lie along the [100] direction; structural units revealed on the free surfaces of the (001) terraces and interpreted as PD are marked out.

A direct STM exploration of the {105} facets confirms the derived model. Being superposed with the empty-state STM image of the cluster {105} facet, it demonstrates an excellent agreement with the experiment (Figure 7b). A typical STM image of the QD facet is presented in Figure 8. Characteristic distances on the facets are as follows: ~10.5 Å in the *<*100*>* directions (along the corresponding side of the base) and ~14 Å in the normal (*<*051*>*) directions. The facets are composed by structural units which are outlined by ellipses in Figure 8a and can be arranged along either [110] or [] direction on the (001) plane. We have interpreted them as pairs of dimers. Their positional relationship is obviously seen in the 3D micrograph presented in Figure 8b.

Dangling bonds of the derived {105}-PD facets, due to high chemical activity, may stimulate Ge atom addition and cluster growth. Thus, less stability and higher activity of the {105}-PD facets compared with the Ge(105)/Si(105)-RS plane, which is usually adopted in the literature for simulation of hut {105} facets, may cause fast completion of hut terraces during epitaxy and be responsible (or even be necessary) for hut formation and growth.

### Cluster density and fractions

**Figure 9:**
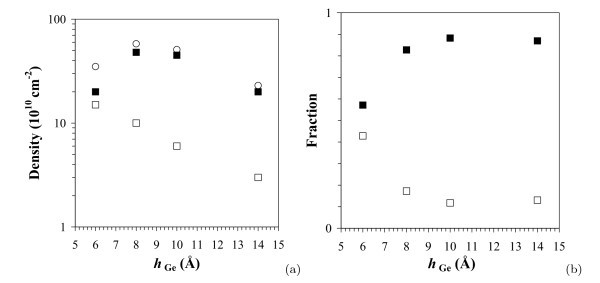
**Density and fractions of the Ge clusters of different species in the arrays formed at *T*_gr _= 360°C**: **(a) **number density and **(b) **fractions; open squares mark the pyramids, black squares designate the wedges, open circles correspond to the total density of huts.

Figure 9a plots the dependence of the cluster density on *h*_Ge _for different clusters in the arrays. It is seen that the density of wedges rises starting from *D*_w _≈ 1.8 *× *10^11 ^cm^-2 ^at the beginning of the 3D growth of Ge (the estimate is obtained by data extrapolation to *h*_Ge _= 5 Å) and reaches the maximum of ~ 5 × 10^11 ^cm^-2 ^at *h*_Ge _~ 8 Å, and the total density of clusters at this point *D*_Σ _~ 6 *× *10^11 ^cm^-2 ^is also maximum. Then, both *D*_w _and *D*_Σ _slowly go down until the 2D growth of Ge starts at *h*_Ge _~ 14 Å and *D*_Σ _≈ *D*_w _~ 2 *× *10^11 ^cm^-2 ^(the contribution of pyramids from *D*_p _to *D*_Σ _becomes negligible--about 3 *× *10^10 ^cm^-2^--at this value of *h*_Ge_). The pyramid density exponentially drops as the value of *h*_Ge _grows (*D*_p _≈ 5 × 10^11 ^exp{2.0 × 10^7 ^*h*_Ge_}; *h*_Ge _is measured in centimetres). The maximum value of *D*_p _≈ 1.8 *× *10^11 ^cm^-2 ^obtained from extrapolation to *h*_Ge _= 5 Å coincides with the estimated initial value of *D*_w_.

The graphs of cluster fractions in the arrays versus *h*_Ge _are presented in Figure 9b. Portions of pyramids and wedges initially very closely similar (~50% at *h*_Ge _~ 5 Å) rapidly become different as *h*_Ge _rises. The content of pyramids monotonically falls. The fraction of the wedge-like clusters is approximately 57% at the early stage of the array growth (*h*_Ge _= 6 Å) and becomes 82% at *h*_Ge _= 8 Å. On further growth of the array, the content of the wedges reaches the saturation at the level of approximately 88% at *h*_Ge _= 10 Å.

It may be inferred from this observation that contrary to the intuitively expected from the consideration of symmetry, the wedge-like shape of the clusters is energetically more advantageous than the pyramidal one, and the more the Ge atoms (and the more the number of terraces) constitute the cluster, the more advantageous it is. The probability of nucleation appears to be close to 1/2 for both wedges-like and pyramidal clusters at the initial stage of the array formation and low growth temperatures. Then, as the array grows, the formation of pyramids becomes hardly probable and most of them, which have already formed, vanish, whereas the nucleation and further growth of wedges continue. The Ge pyramids on the Si(001) surface turned out to be less stable objects than the wedges.

**Figure 10:**
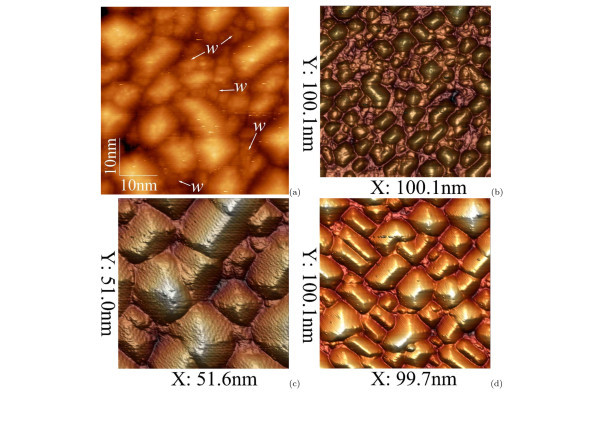
**STM 2D and 3D micrographs of Ge hut cluster dense arrays at different coverages (*T*_gr _= 360°C)**: **(a, b) ***h*_Ge _= 8 Å [(a) 50.6 × 49.9 nm, *w *is WL, *U*_s _= +2.0 V, *I*_t _= 80 pA; (b) *U*_s _= +2.0 V, *I*_t _= 100 pA]; (c, d) *h*_Ge_ = 10 Å [ *U*_s _= +2.1 V, *I*_t _= 100 pA].

**Figure 11:**
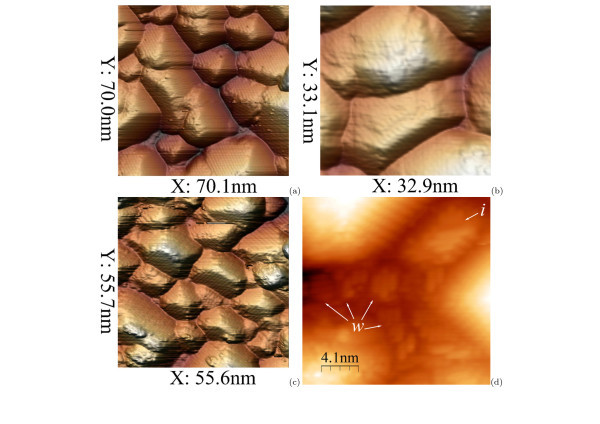
**STM topographs of Ge hut cluster dense arrays at different coverages (*T*_gr _= 360°C)**: **(a, b) ***h*_Ge _= 14 Å [**(a) ***U*_s _= +1.75 V, *I*_t _= 80 pA, **(b) ***U*_s_ = +3.0 V, *I*_t _= 100 pA]; **(c, d) ***h*_Ge _= 15 Å [**(c) ***U*_s _= +2.0 V, *I*_t _= 120 pA, **(d) **20.3 *× *20.4 nm, *U*_s_ = +3.6 V, *I*_t _= 120 pA]; *w *indicates the WL patches, and *i *shows a distorted small Ge island 3 ML high over WL.

Notice also that at *T*_gr _= 360°C and the flux of Ge atoms d*h*_Ge _/ d*t *= 0.15 Å/s, the point *h*_Ge _= 10 Å is particular. Not only the fraction of pyramids saturates at this point but the array overall has the most uniform sizes of the clusters composing it (Figures 4, 10 and 11). We concluded this not only on the basis of analysis of the STM images of the Ge/Si(001) arrays but also from the data of the Raman scattering by the Ge/Si heterostructures with different low-temperature arrays of Ge QDs [42,43]. We refer to such arrays as optimal.

### Array life cycle

A qualitative model accounting for the presence of the particular point at the low-temperature array growth is simple. The case is that at low enough temperatures of the array growth, the new Ge cluster nucleation competes with the process of growth of earlier formed clusters. The height of the dominating wedge-like clusters is observed to be limited by some value depending on *T*_gr_.^f ^At small *h*_Ge_, Ge clusters are small enough, and the distances between them are large enough compared with the Ge atom (or dimer) diffusion (migration) length on the surface for nucleation of new clusters on the Ge WL in the space between the clusters (Figures 4 and 10a, b). At *h*_Ge _= 10 Å and the above d*h*_Ge _/ d*t *values, the equilibrium of parameters (cluster sizes and distances between them, diffusion length at given temperature, Ge deposition rate, etc.) sets in, the rate of new cluster nucleation is decreased, and the abundant Ge atoms are mainly spent to the growth of the available clusters (Figure 10c, d). After the clusters reach their height limit and in spite of it, Ge atoms continue to form up their facets. As soon as the most of the clusters reach the height limit, nucleation of new clusters becomes energetically advantageous again, and the nucleation rate rises. The second phase of clusters appears on the WL and fills whole its free surface as *h*_Ge _is increased (Figure 11). Further increase of *h*_Ge _results in 2D growth mode. It is clear now why the array is the most homogeneous (optimal) at *T*_gr _= 360°C and *h*_Ge _= 10 Å, whereas the dispersion of the cluster sizes is increased at higher and lower values of *h*_Ge _because of the small clusters contained in the array. It is clear also that the optimal array will appear at different values of *h*_Ge _when *T*_gr _or d*h*_Ge _= d*t *are different.

As it follows from the data presented in this section and the section 'Array and hut cluster nucleation', the Ge hut array evolution and life cycle goes through three main phases: at *T*_gr _= 360°C, the array nucleates at *h*_Ge _~ 5 Å (Figure 1), it reaches ripeness and optimum to *h*_Ge _~ 10 Å (Figure 10) and finishes its evolution at *h*_Ge _~ 14 Å by filling the entire surface (Figure 11). Most of the clusters start coalescing (Figure 11b), and 2D growth begins at greater *h*_Ge _(Figure 11c).

Nevertheless, free areas of WL still remain even at *h*_Ge _= 15 Å (Figure 11d). The structure of the parches ('*w*') remains the same as in the beginning of the array formation although the WL regions are surrounded by large huts. Small 3D islands ('*i*'), although very distorted, are still recognizable on WL between the large huts. The hut nucleation on WL goes on even at as high coverages as 15 Å, when virtually total coalescence of the mature huts have already happened.

## Conclusion

In summary, we have studied the array nucleation phase and identified the nuclei of both hut species, determined their atomic structure, and observed the moment of appearance of the first generation of the nuclei on WL. We have investigated with high spatial resolution the peculiarities of each species of huts and their growth and derived their atomic structures. We have concluded that the wedge-like huts form due to a phase transition reconstructing the first atomic terrace of the growing cluster when dimer pairs of its second atomic layer stack up; the pyramids grow without phase transitions. In addition, we have concluded that wedges contain vacancy-type defects on the penultimate terraces of their triangular facets which may decrease the energy of addition of new atoms to these facets and stimulate the quicker growth on them than on the trapezoidal ones and rapid elongation of wedges. We have shown also, comparing the structures and growth of pyramids and wedges, that shape transitions between them are impossible. And finally, we have explored the array evolution during MBE right up to the concluding phase of its life when most clusters coalesce and start forming a nanocrystalline 2D layer.

## Abbreviations

CMOS: complementary metal-oxide semiconductor; IR: infrared; MBE: molecular beam epitaxy; UHV: ultra-high vacuum; STM: scanning tunneling microscope; ML: monolayer; QD: quantum dot; WL: wetting layer; PD: pairs of dimers; RS: rebonded step.

## Competing interests

The authors declare that they have no competing interests.

## Authors' contributions

LA participated in the design of the study, carried out the experiments, performed data analysis, took part in discussions and interpretation of the results; she proposed the structural models. VY conceived of the study and designed it, processed images and performed data analysis, took part in discussions and interpretation of the results; he also supervised and coordinated the research projects.
